# Supplementary material for: Complex‐centric proteome profiling by SEC‐SWATH‐MS
Source: Mol Syst Biol. 2019 Jan 14;15(1):e8438. doi: 10.15252/msb.20188438 (PMC6346213; doi:10.15252/msb.20188438)
Supplement: Supplementary file 7 — Dataset EV6 [file MSB-15-e8438-s007.zip › feature_plots_bioplex/P35613.pdf]

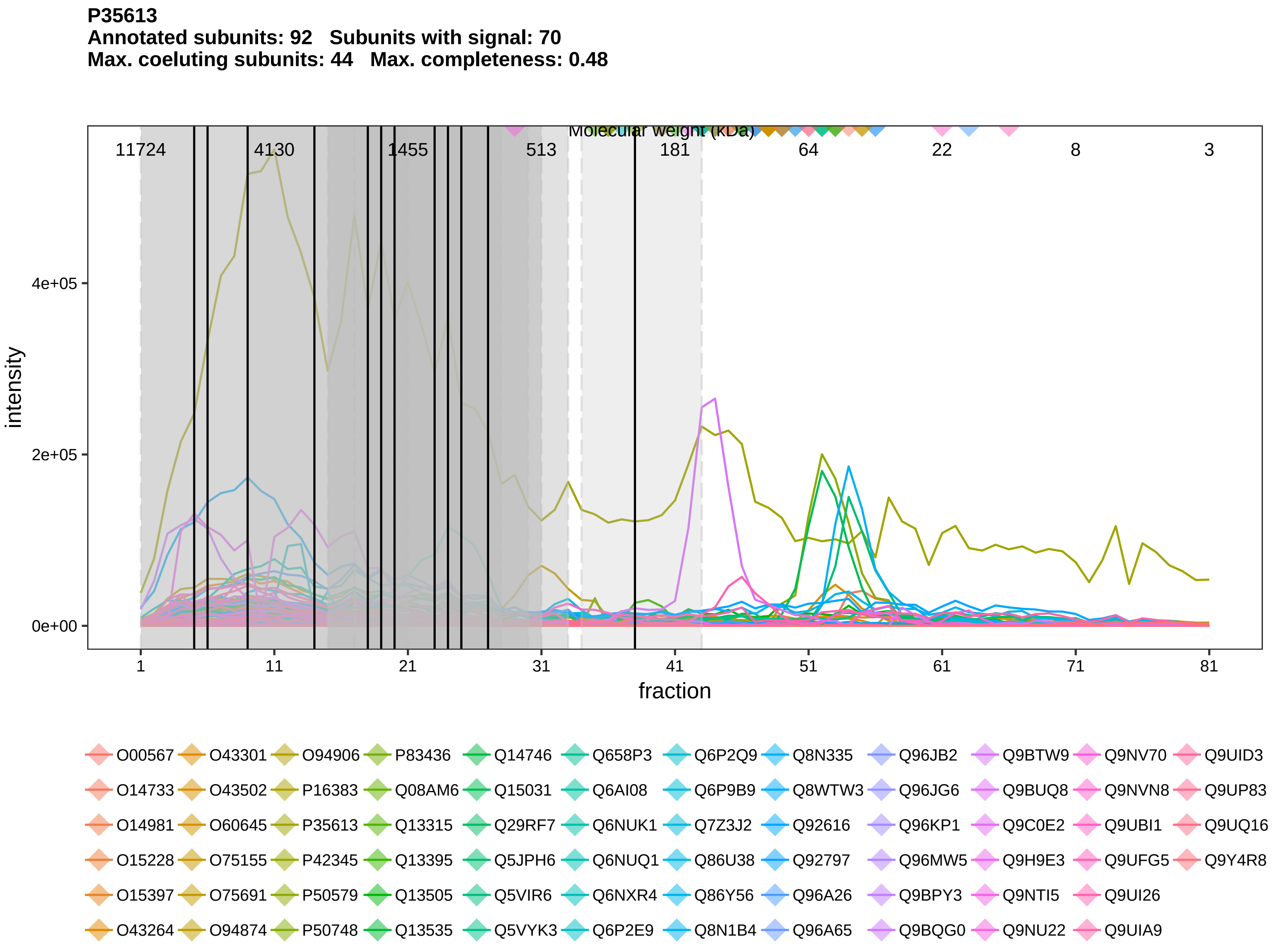
P35613
Annotated subunits: 92 Subunits with signal: 70
Max. coeluting subunits: 44 Max. completeness: 0.48
Molecular weight (kDa)
11724
4130
1455
513
181
64
22
8
3
intensity
4e+05
2e+05
0e+00
1
11
21
31
41
51
61
71
81
fraction
O00567 O43301 O94906 P83436 Q14746 Q658P3 Q6P2Q9 Q8N335 Q96JB2 Q9BTW9 Q9NV70 Q9UID3
O14733 O43502 P16383 Q08AM6 Q15031 Q6AI08 Q6P9B9 Q8WTW3 Q96JG6 Q9BUQ8 Q9NVN8 Q9UP83
O14981 O60645 P35613 Q13315 Q29RF7 Q6NUK1 Q7Z3J2 Q92616 Q96KP1 Q9C0E2 Q9UBI1 Q9UQ16
O15228 O75155 P42345 Q13395 Q5JPH6 Q6NUQ1 Q86U38 Q92797 Q96MW5 Q9H9E3 Q9UFG5 Q9Y4R8
O15397 O75691 P50579 Q13505 Q5VIR6 Q6NXR4 Q86Y56 Q96A26 Q9BPY3 Q9NTI5 Q9UI26
O43264 O94874 P50748 Q13535 Q5VYK3 Q6P2E9 Q8N1B4 Q96A65 Q9BQG0 Q9NU22 Q9UIA9
